# Reassessing the feasibility of the zygote score for predicting embryo viability in IVF/ICSI using the GnRH antagonist protocol compared to the long protocol

**DOI:** 10.1371/journal.pone.0171465

**Published:** 2017-02-02

**Authors:** Pin-Yao Lin, Fu-Jen Huang, Fu-Tsai Kung, Yi-Chi Lin, Hsin-Ju Chiang, Yu-Ju Lin, Kuo-Chung Lan

**Affiliations:** 1 Department of Obstetrics and Gynecology, Kaohsiung Chang Gung Memorial Hospital and Chang Gung University College of Medicine, Kaohsiung, Taiwan; 2 Graduate Institute of Clinical Medical Sciences, Chang Gung University, Kaohsiung, Taiwan; Peking University Third Hospital, CHINA

## Abstract

**Background:**

Many factors from the oocyte/sperm or the process of fertilization may affect the zygote formation. The zygote score (Z-score) describes the quality of a human zygote based on its pronuclear morphology, nucleolar precursor bodies, and alignment of polar bodies, and it can be used in the selection process at the zygote stage for embryo transfer or cryopreservation.

**Objective:**

The aim of this retrospective cohort study was to investigate the relationship between different controlled ovarian stimulation (COS) protocols and the zygote score (Z-score) and to assess the feasibility of the Z-score for predicting embryo survival in the GnRH-antagonist (GnRH-ant) protocol.

**Methods:**

It is a retrospective, single-center cohort study. A total of 3,826 zygotes with normal fertilization were analyzed from 744 in vitro fertilization /intra-cytoplasmic sperm injection (IVF/ICSI) cycles (long protocol n = 392; GnRH-ant n = 352) between Jan 2010 and April 2014 in the IVF unit of Chang-Gung Memorial Hospital Kaohsiung Medical Center.

**Results:**

The Z-score distribution differed significantly between these two protocols. The overall Z-score was poorer for zygotes from GnRH-ant cycles (p<0.05). Univariate and multivariate analyses indicated the type of COS protocol is one of the main determinants of Z-score grading. Our study found good-quality day 3 embryo/blastocyst formation and the cumulative embryo survival rate were correlated with the Z-score but not the COS protocol. With the GnRH-ant protocol, the number of Z1 in the transferred cohort embryos was significantly correlated with the clinical pregnancy rate (r = 0.976; p = 0.024) and live birth rate (r = 0.971; p = 0.029). This correlation was not seen with the long protocol.

**Conclusions:**

The Z-score distribution for the GnRH antagonist cycles was poorer than that of the long protocol, but the Z-score system is a valuable parameter for predicting embryo viability in the GnRH-ant protocol, providing a strong correlation with the clinical pregnancy rate and live birth rate.

## Introduction

Clinicians have used the GnRH-ant protocol for assisted reproduction in recent decades as an alternative to the GnRH agonist (GnRH-ag) protocol [1–3]. Recent researches have shown that the use of GnRH antagonist is associated with decreasing the risk of severe ovarian hyperstimulation syndrome without reducing live birth rate. [4–7]. However, the impact of ovarian stimulation on oocyte and embryo quality is still unclear.[8]

The zygote score (Z-score) is an established scoring system based on the pronuclear morphology, nucleolar precursor bodies, and alignment of polar bodies in the human zygote at approximately 16 to 18 h after insemination [9]. Scoring of zygotes has proven to be useful for selection at the zygote stage for embryo transfer or cryopreservation, especially in countries with legal restrictions [10, 11].Although numerous studies have associated positive clinical results with the use of the Z-score for embryo selection [9, 12–14], other studies did not find the Z-score to be a valuable predictor [15–17]. When reviewing the literature describing the use of the Z-score in morphologic selection [18], most studies analyzed the Z-score in the GnRH-ag protocol, without considering the feasibility of using the Z-score for the GnRH-ant protocol. The importance of zygote scoring with the gonadotropin-releasing hormone antagonist (GnRH-ant) protocol has not been established.

The present retrospective study examined a cohort of embryos derived from patients undergoing in vitro fertilization/intra-cytoplasmic sperm injection (IVF/ICSI). The aim of this study was to determine the relationship between the different controlled ovarian stimulation (COS) protocols and the Z- score and to determine the feasibility of using the Z-score to predict embryo survival in the GnRH-ant protocol.

## Materials and methods

### Patients

This retrospective study included 3,826 zygotes with normal fertilization from 643 women and followed 744 IVF/ICSI cycles at the Chang-Gung Memorial Hospital Kaohsiung Medical Center between January 1, 2010 and April 30, 2014. All of the women had cycles from COS with a luteal phase down-regulated protocol (long protocol) or a GnRH-ant protocol, and all couples had at least one zygote with two pronuclear (PN) formations after insemination/ICSI at 16 to 18 h. All cases with unsuccessful oocyte retrieval or polyspermic zygotes were excluded. Women were excluded if they underwent COS with natural cycles or clomiphene using an ultra-long protocol or a short protocol. All charts were retrospectively reviewed by one physician. The study was approved by an appropriately constituted the Institutional Review Board of the Ethics Committee of Chang Gung Memorial Hospital (Institutional Review Board Number: 201600478B0) and Institutional Review Board waived the need for consent. This research did not receive any specific grant from funding agencies in the public, commercial, or not-for-profit sectors.

### Controlled ovarian stimulation, oocyte retrieval, oocyte preparation, embryo culture, assessment of fertilization, zygote score, and embryo grading

COS, oocyte retrieval, embryo culture, and embryo transfer were performed according to our previously described protocols [12, 19]. The long protocol and GnRH-ant protocol for COS were individualized and depended on ovarian reserve, age, baseline serum follicle stimulating hormone (FSH) concentration, and previous response to COS. Briefly, women received the long protocol with pituitary down-regulation using leuprolide acetate (Lupron®; Takeda, Tokyo, Japan) with the initial dose of gonadotropin, either human menopausal gonadotropin (hMG) or FSH (purified or recombinant), individualized for each patient (range: 150 to 300 IU). Further dose adjustments were made according to the individual’s ovarian response, based on the serum estradiol (E2) level and ultrasonographic monitoring of follicular growth. When the lead follicle was 16–18 mm in diameter, leuprolide acetate and FSH were discontinued, and human chorionic gonadotropin (hCG) (Ovidrel®; Serono, Modugno, Italy) was administered. Oocyte retrieval was performed by transvaginal ultrasound-guided follicle aspiration at 36–38 h after hCG administration. In the GnRH-ant protocol, patients were given gonadotropin stimulation and then suppression using a flexible GnRH-ant protocol (Ganirelix acetate: 0.25 mg, MSD; Cetrorelix acetate: 0.25 mg; Serono) when the leading follicle was 14 mm. When 2 more follicles reached diameters of 17 mm, a 6,500 IU dose of hCG (Ovidrel®; Serono, Modugno, Italy) was administered. Oocyte retrieval was performed 36–38 h later by transvaginal aspiration under ultrasound guidance. Oocytes were graded for maturity on the basis of the morphological characteristics of the cumulus mass, corona radiata, ooplasm, and detached membrane granulose cells. [20] Standard IVF/ICSI procedures were used for oocyte fertilization, as previously described [12]. Fertilization was evaluated 16–18 h after IVF or ICSI. A zygote with two pronuclei (2PN) was defined as normal fertilization. All zygotes were scored according to the modified Scott scoring system [21]. A single team of embryologists coordinated all procedures of IVF laboratory to ensure that the culture processes and the embryo assessments were standardized.

For PN scoring, zygotes were divided into 4 categories (Z1-Z4) based on 3 major features: size and location of the nuclei, appearance of the cytoplasm, and numbers, sizes, and distribution patterns of nucleolar precursor bodies within the nuclei. (S1 Fig)) Z1 zygotes have an equal number of nucleoli aligned at the PN junctions. Z2 zygotes have an equal number and size of nucleoli (3 to 7) that are equally scattered in the 2 PNs, but the nucleoli have not yet aligned at the PN junction. Z3 zygotes are characterized by inequality of the nuclei (unequal size, unequal numbers, or unequal alignment at the PN junction). Z4 zygotes have PN that are separated or different in size and small nucleoli that are partially aligned or scattered[21].

Embryos were cultured on days 1 to 3 in G1 ^TM^ medium (Scandinavian IVF Science) and on days 3 to 5 in G2 ^TM^ medium (Scandinavian IVF Science). Veeck’s morphological grading system [22] was adopted for day-3 embryo scoring. A “good” 3-day embryo was defined as one that had a Veeck’s grade of 1, with 8 cells, blastomeres of equal size, and no cytoplasmic fragments. Embryos were assessed by survival, morphology, and rate of cleavage. Embryos that had the same number of blastomeres at two sequential observations and zygotes that remained blocked at the pronuclear stage were classified as “developmentally arrested”. Embryos were transferred on day 2, 3, 4, 5 or 6 after oocyte retrieval, as appropriate for each individual. The risk of embryo arrest was defined as the time to a first event. The start point for determining the duration of embryo survival was the date when PN appeared after 16–18 h of incubation, and the end point was after 5 days of extended culture. The study endpoints were the duration of overall survival and event-free survival (*i*.*e*. embryo arrest). In the analysis of embryo arrest, data from patients who received a day-2, day-3, day-4, day-5 or day-6 embryo transfer were censored after the time of transfer (loss due to transfer) or cryopreservation. Luteal phase support continued until the day pregnancy was confirmed by detection of hCG in the urine. If conception occurred, micronized progesterone supplementation was provided for an additional 4 weeks. Clinical pregnancy was defined as the presence of an intrauterine gestational sac with positive cardiac movement on ultrasound [23]. The live birth rate per transfer was defined as the proportion of IVF cycles reaching embryo transfer that resulted in the birth of at least one live-born child.

### Statistical analysis

Continuous data are given as the mean ± standard deviation (SD). The Mann-Whitney rank sum test was used to compare continuous data, and the χ^2^ test or Fisher’s exact test was used to compare categorical variables. A Pearson correlation coefficient (*r*) was calculated for correlations. All statistical analyses were performed with SPSS, version 17.0 (SPSS, Inc., Chicago, IL, USA). All *p* values were two-sided, and a *p* value less than 0.05 was considered statistically significant. Logistic regression analysis was used to determine the significance of factors associated with a Z-score of 1 (Z1). Multivariate logistic regression analysis was then performed to further examine factors associated with Z1, considering the following variables: female age, male age, ICSI or IVF, etiology of infertility, body mass index, COS protocol, days of stimulation, FSH dosage, E2 level on the day of hCG detection, E2 level per oocyte, P4 level, maturity of oocyte, and number of same-cohort oocytes. Cumulative survival rates were calculated by life-table analysis using the Kaplan–Meier product limit procedure at each day. The differences between groups with different Z-scores were assessed using the log-rank test. A *p*-value less than 0.05 was considered significant [24].

## Results

### General characteristics

During the period, 744 fresh cycles from 643 women were included, which consisted of 352 long protocol cycles and 392 GnRH-ant protocol cycles. Table 1 shows the characteristics of these cycles.

**Table 1:** Characteristics of the 744 IVF/ICSI Cycles.

Characteristic	N, mean ± SD, or % (range)
No. of cycles with at least one zygote	744
No. of infertile couples	643
No. of cycles of long protocol	352
No. of cycles of GnRH-ant protocol	392
No. of IVF cycles	486
No. of ICSI cycles	258
Age of females (years)	34.9 ± 4.9
Age of males (years)	37.8 ± 5.63
Body mass index (kg/m^2^)	22.6 ± 9.47
Infertility	
Primary	487
Secondary	257
Duration of infertility (years)	4.21 ± 3.98
No. of oocytes retrieved	6.77 ± 4.14
Endometrial thickness on hCG day (cm)	1.35 ± 1.2
Estradiol (pg/mL) on hCG day	2300 ± 1553
Progesterone (ng/mL) on hCG day	1.00 ± 0.55
Indication	
Tubal factor	186
Male factor	159
Endometriosis	72
Ovulatory factor	78
Unexplained	92
Combined factors	159
Normal fertilization rate (%)	76% (3826/5037)
Mean no. of embryos transferred	2.19 ± 0.86
No. of cycles on day-2/day-3/day-4/day-5/day-6 transfer	7/380/82/244/2
Clinical pregnancy rate on day-2/day-3/day-4/day-5/day-6 transfer (%)	28.6%/31.8%/31.7%/50%/50%
Overall clinical pregnancy rate/transfer cycle (%)	38.0% (272/715)
Implantation rate (%)	23.2% (378/1629)

**Fig 1:**
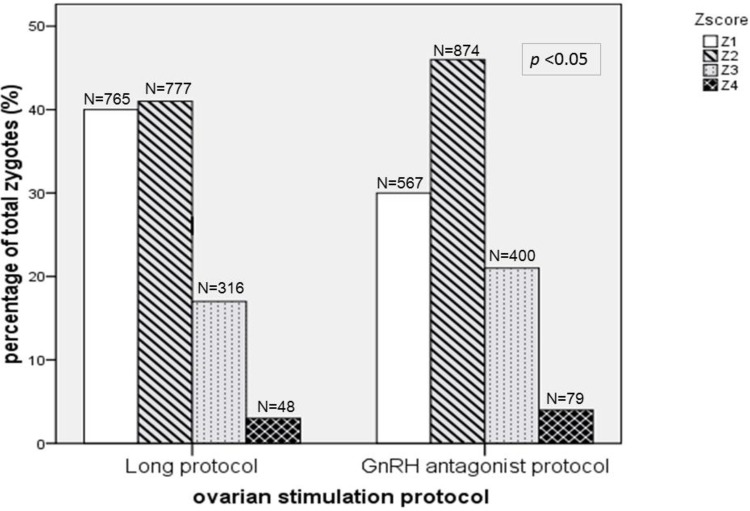
Overall distribution of zygote‐score for 3826 zygotes in 744 cycles from the long protocol (n = 352) and the GnRH-ant protocol(n = 392).

A total of 3,826 zygotes with normal fertilization produced from 744 fresh cycles following IVF/ICSI were examined. We performed fertilization check using the Z-score, a system that has been used to evaluate fertilization in our center since 2001. Fig 1 shows the distribution of Z-scores. Most zygotes in the long protocol had scores of Z1 and Z2. (N/ (%); Z1: 765(40.1%), Z2: 777(40.8%), Z3:316(16.6%), Z4:48(2.5%)) Fewer zygotes in the GnRH-ant protocol had scores of Z1, and more had scores of Z2. (N/ (%); Z1: 567(29.5%), Z2:874(45.5%), Z3:400(20.8%), Z4:79(4.1%)). The Z-score distributions in the two groups were significantly different (*p* < 0.05) and shifted toward poorer Z-scores in the GnRH-ant protocol.

### Factors that affect the Z-score

In this retrospective and observational study, we did not perform statistical comparisons between cycles from the different protocols due to the risk of selection bias. Instead, we observed the cohort zygotes from the two protocols and analyzed potential factors that may influence the zygote score. Our previous study indicated good (Z-1) zygote had higher implantation potential.[12]We further analyzed the clinical and laboratory factors associated with a good Z-score of Z1. Univariate analyses (Table 2) showed that a score of Z1 correlated significantly with the COS protocol used (*p* < 0.001), oocyte maturity (*p* < 0.001), progesterone on the day of hCG detection (*p* < 0.001), E2 concentration per oocyte (*p* = 0.028), total FSH dosage (*p* = 0.025), and duration of FSH stimulation (*p* = 0.028).

**Table 2:** Factors Affecting the Zygote with Good Zygote score (Z-1): Univariate Analysis.

Variable	% all Z1 zygotes	*p* value
**COS protocol**		
Long protocol vs. GnRH-ant protocol	57.3% vs. 42.7%	**<0.001** ^**a**^
**Maturity of oocytes**		
Mature vs. Non-mature	91.7% vs. 8.3%	**<0.001**^**a**^
**Progesterone on hCG day (ng/mL)**		
≦1.0 vs. >1.0	45.7% vs. 54.3%	**0.004** ^**a**^
**E2 (pg/mL)/oocyte**		
≦376 vs. >376	65.8% vs. 34.2%	**0.028** ^**a**^
**Ampoules of 75 IU FSH**		
≦30 vs. >30	52.7% vs. 47.3%	**0.025** ^**a**^
**Days of FSH stimulation**		
≦ 8.92 vs. >8.92	42.9% vs. 57.1%	**0.028** ^**a**^
**Fertilization method**		
IVF vs. ICSI	64.9% vs. 35.1%	0.091
**Body mass index (kg/m**^**2**^**)**		
≦22.6 vs. >22.6	61.6% vs. 38.4%	0.148
**Age of female partner (years)**		
≦34.9 vs. >34.9	57.8% vs. 42.2%	0.051
**Age of male partner (years)**		
≦37.8 vs. >37.8	61.3% vs. 38.7%	0.053
**No. of cohort oocytes retrieved**		
≦6.7 vs. >6.7	29.1% vs. 70.9%	0.578
**No. of cohort mature oocytes**		
≦3.24 vs. >3.24	34.4% vs. 65.6%	0.057
**Cohort 2PN No.**		
≦5.04 vs. >5.04	35.4% vs. 64.6%	0.099
**Primary/second infertility**		
Primary vs. Secondary	64.2% vs. 35.8%	0.696
**Duration of infertility (years)**		
≦4.2 vs. >4.2	69.4% vs.30.6%	0.883
**E2 (pg/mL) on hCG day**		
≦2300 vs. >2300	40.4% vs. 59.6%	0.504
**Endometrial thickness on hCG day (cm)**		
≦1.35 vs. >1.35	56.9% vs. 43.1%	0.053

^a^ A *p-*value less than 0.05 was considered statistically significant

We then used multivariable logistic regression analysis to identify factors associated with a score of Z1 (Table 3). These results indicate that a score of Z1 was significantly and independently associated with oocyte maturity (*p* < 0.001), number of cohort 2PN (*p* < 0.001), and COS protocol used (*p* < 0.001). The observation of significantly fewer Z1 zygotes from the GnRH-ant protocol is compatible with our clinical observations.

**Table 3:** Factors Affecting the Zygote with Good Zygote score (Z-1): Multivariable Regression Analysis.

Variable	B	SEM	Wald	P value	Exp(B)	95% CI
Oocyte maturity	2.654	0.124	459.451	<0.0001 *	14.212	11.149,18.1115
Cohort 2PN No.	-0.597	0.129	21.444	<0.0001 *	0.551	0.428,0.709
COS protocol	-0.581	0.097	35.594	<0.0001 *	0.560	0.462,0.677
No. of cohort oocytes retrieved	0.177	0.173	1.052	0.305	1.194	0.851,1.675
Age of female (years)	-0.002	0.104	0.007	0.983	0.998	0.814,1.223
No. of cohort mature oocytes	0.262	0.167	2.467	0.116	1.300	0.937,1.803
Age of male (years)	-0.009	0.100	0.009	0.926	0.991	0.815,1.215
Body mass index (kg/m^2^)	-0.148	0.093	2.569	0.109	0.862	0.719,1.034
Fertilization method	0.130	0.094	1.912	0.167	1.139	0.947,1.371
Primary/second infertility	0.087	0.093	0.876	0.349	1.091	0.909,1.310
Duration of infertility (years)	-0.025	0.097	0.064	0.800	0.976	0.807,1.180
E2 (pg/mL) on hCG day	0.154	0.117	1.733	0.188	1.166	0.928,1.466
Progesterone on hCG day(ng/mL)	-0.115	0.094	1.503	0.220	0.891	0.742,1.071
E2 (pg/mL)/oocyte	-0.063	0.099	0.402	0.526	0.939	0.773,1.140
Ampoules of 75 IU FSH	-0.127	0.098	1.660	0.198	0.881	0.727,1.068
Endometrial thickness on hCG day (cm)	-0.127	0.090	1.999	0.157	0.881	0.739,1.050
Days of FSH Stimulation	-0.004	0.104	0.002	0.969	0.996	0.813,1.220

B: intercept; SEM: standard error of the mean; Wald: Wald statistic; Exp (B): adjusted odds ratio; CI: confidence interval.

* A *p-*value less than 0.05 was considered statistically significant

### Relationship of Z-score and day-3 good embryo/blastocyst formation/embryo survival

**Fig 2:**
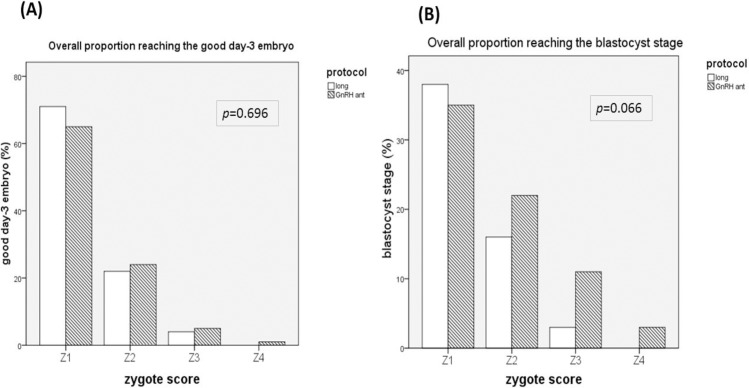
Relationship between the Z- score with the formation of good quality embryos on day 3 (A) and blastocysts on day 5 or 6 (B) following the long protocol and the GnRH-ant protocol.

**Fig 3:**
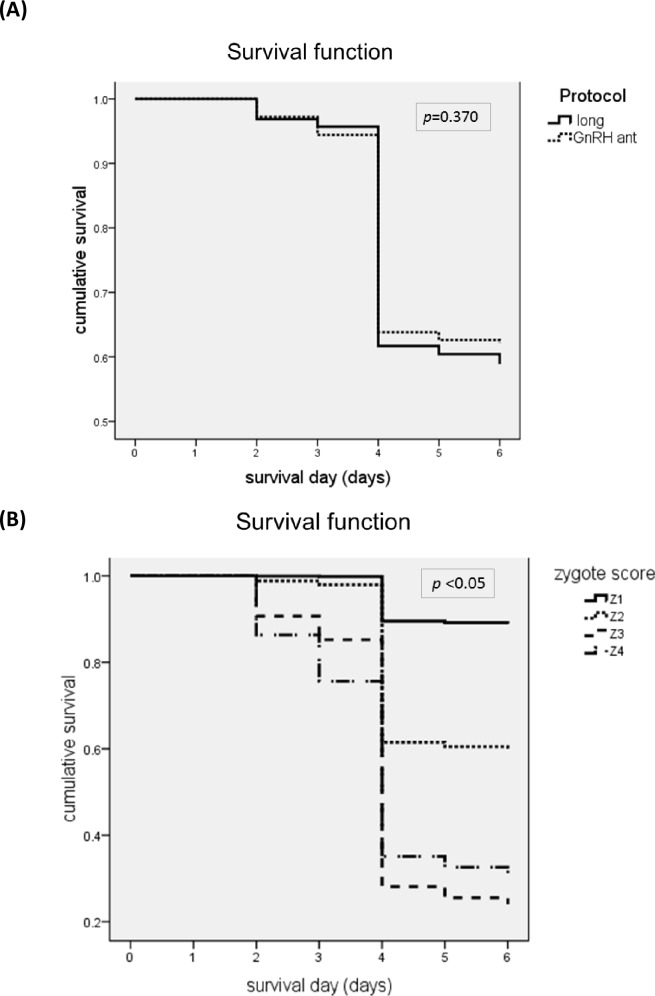
Overall day-5 arrest‐free survival of zygotes resulting from the long protocol (solid line) and the GnRH-ant protocol (dotted line) (A) and of zygotes with different Z-scores (B).

The relationships between the Z-score and good day-3 embryo and blastocyst formation were investigated.(Fig 2A and 2B) The results indicated that the Z-score had similar correlations with good embryo formation on day-3 for both protocols. Long protocol vs. GnRH-ant: (N/ (%); Z1: 539(70.6%) *vs*. 370(65.3%), Z2: 174(22.4%) *vs*. 208(23.7%), Z3: 13(4.1%) *vs*. 21(5.2%), Z4: 0(0%) *vs*. 1(1%); *p* = 0.696) (Fig 2A). An analysis of the relationship between Z-score and blastocyst formation also indicated similar correlations for the two protocols. Long protocol vs. GnRH-ant (N/ (%); Z1:288(38%) *vs*. 198(35%), Z2:123(16%) *vs*. 194(22%), Z3: 9(3%) *vs*. 45(11%), Z4:0(0%) *vs*. 2(3%); *p* = 0.066) (Fig 2B). Furthermore, we tested the feasibility of using the Z-score in the GnRH-ant protocol to predict embryo survival on day 5 (Fig 3A and 3B). The overall survival rates for the long protocol and GnRH-ant protocol were similar (59 ± 1% *vs*. 62 ± 1%, *p* = 0.370) (Fig 3A). As expected, the overall survival rate on day 5 declined as the Z-score increased (Z1: 89 ± 1%, Z2: 60 ± 1%, Z3: 24 ± 2%, Z4:29 ± 4%; *p* <0.05; Fig 3B).

**Fig 4:**
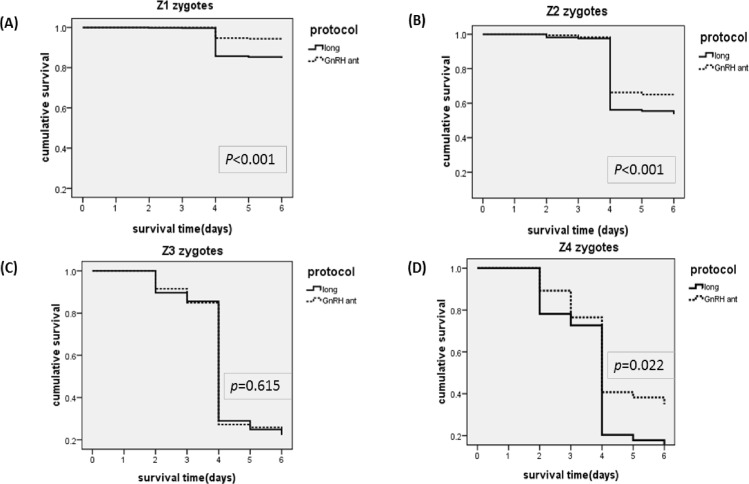
Day-5 arrest‐free survival of zygotes resulting from the long protocol (solid line) and the GnRH-ant protocol (dotted line) with scores of Z1 (A), Z2 (B), Z3 (C), and Z4 (D).

Cumulative survival rates were compared for the long protocol and GnRH-ant protocol for zygotes with different Z-scores (Fig 4A–4D). The GnRH-ant protocol led to better survival for Z1 zygotes (85 ± 1% *vs*. 94 ± 1%, *p* < 0.001)(Fig 4A), Z2 zygotes (54 ± 2% *vs*. 65 ± 2%, *p* < 0.001) (Fig 4B), and Z4 zygotes (14 ± 5% *vs*. 35 ± 4%, *p* = 0.022) (Fig 4D), but not for Z3 zygotes (22 ± 3% *vs*. 25 ± 2%, *p* = 0.615) (Fig 4C).

Thus, our study found that good-quality day 3 embryo/blastocyst formation and the cumulative survival rate were correlated with the Z-score but not the COS protocol.

### Relationship of Z-score and pregnancy outcomes

**Fig 5:**
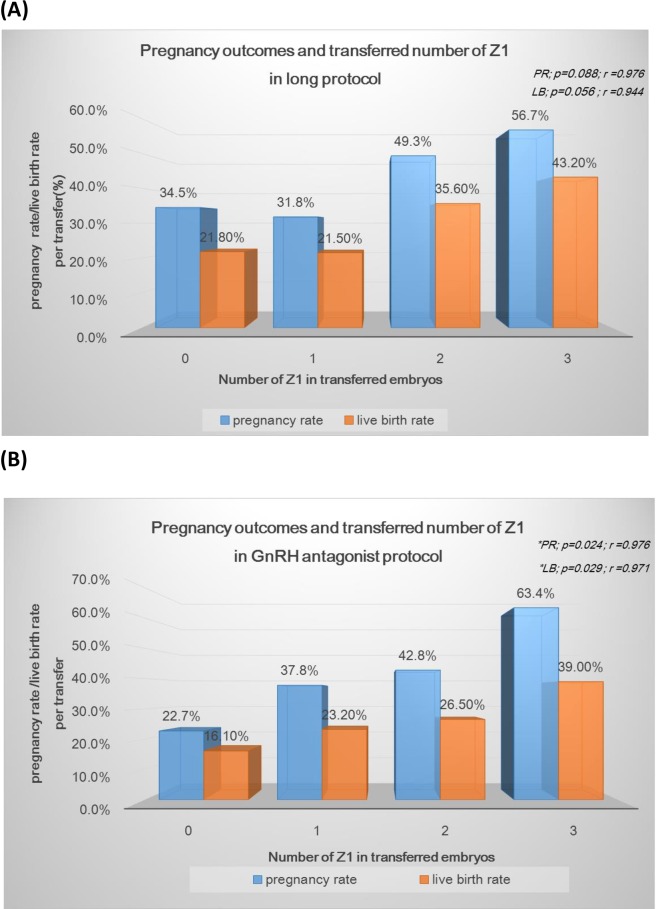
The correlation between the number of transferred good zygote (Z1) and pregnancy outcomes in long protocol (A) and GnRH-ant (B).

Fig 5A and 5B displays the relationships between the number of good zygotes (Z1) transferred and pregnancy outcomes in the long (A) and GnRH-ant (B) protocols. In our study, a mean of 2.19 ± 0.86 embryos were transferred for each woman; therefore, it is difficult to compare the pregnancy outcomes according to Z-score based on single embryo transfer. Instead of single embryo transfer, we compared the number of good zygotes (Z1) transferred and pregnancy outcomes. For the long protocol, the number of Z1 in the transferred cohort embryos was not significantly correlated with the clinical pregnancy rate (*r* = 0.976; *p* = 0.088) or live birth rate (*r* = 0.944; *p* = 0.056). For the GnRH-ant protocol, the number of Z1 in the transferred cohort embryos was significantly correlated with the clinical pregnancy rate (*r* = 0.976; *p* = 0.024) and live birth rate (*r* = 0.971; *p* = 0.029).

## Discussion

To our knowledge, the current study is the first to report the effect of different COS protocols on zygote morphology and the feasibility of using the Z-score to predict embryo survival in the GnRH-ant protocol.

Zygote formation follows the dramatic reorganization of sperm chromatin by many factors stored within the oocyte [25, 26]. The morphological characteristics of the zygote indicate gamete quality and the potential for subsequent embryo implantation [27, 28]. Zygotes with unequal numbers or sizes of nucleoli likely display asynchrony between male and female pronucleus development [29, 30].

Many factors from the oocyte/sperm or the process of fertilization may affect the Z-score. Our IVF laboratory adopted a modified Scott scoring system beginning in 2001 to monitor fertilization. Our previous report (Lan, 2003) indicated that the Z‐score is an additional criterion that can be used to select embryos for extended culture and is useful when selecting embryos for transfer. However, the distribution of Z‐scores changes significantly when the GnRH-ant protocol is used for COS. In particular, we found that the Z-scores from the GnRH-ant protocol were generally poorer than those from the long protocol. Significantly fewer Z1 zygotes resulted from the GnRH-ant protocol, which is compatible with our clinical observations.

Some possible explanation for the differences in the Z-score between the COS protocols involves the different endocrine profiles during follicular growth. First, the long protocol induces profound suppression and leads to simultaneous maturation of antral follicles [8, 31, 32]. Therefore, more mature follicles are recruited, and typically more Z1 zygotes are obtained. In contrast, the GnRH-ant protocol generates a more natural pattern of follicular recruitment, with uneven follicle sizes and fewer mature oocytes. Second, previous studies have suggested that the COS protocols have differing effects on ovarian E2 metabolism [33, 34] and may affect follicular growth and/or luteal function. More specifically, Khalaf *et al*. [32] found that the protein kinase C (PKC) pathway was desensitized in GnRH-ag-treated granulosa cells, and this increased the levels of FSH and cAMP-mediated steroidogenesis. Third, in addition to the different endocrine profiles between the two protocols, the effect of the GnRH receptor on the oocyte/zygote should also be considered. GnRH receptors are expressed in the follicles at the gonadotropin-sensitive stage and in luteal cells. The correlation between the expression of GnRH receptors and follicular stage suggests that GnRH receptors directly influence folliculogenesis and oocyte development [35, 36], but the mechanism by which GnRH affects the ovary is not completely understood. Thus, when GnRH-ant is used in IVF cycles, it has an unclear effect on the morphology and quality of oocytes. Animal studies indicate that GnRH-ant may have an adverse effect on primordial follicle survival in some species [37], but only a few studies examined the effect of GnRH-ant on human oocytes and embryos, with inconsistent results [38–40]. The results of our study also indirectly support the hypothesis that GnRH receptors play a role in oocyte maturation and affect zygote formation.

Although more mature oocytes/Z-1 zygotes were produced from long protocol than GnRH-ant possibly because of better endocrine profiles and no effect of GnRH antagonist; however, the overall Z-1 zygotes from long protocol had poorer survival and not correlated with clinical pregnancy. Our results suggested that long protocol only improved morphological maturity of oocytes and zygotes’ morphology without changing the survival potential of oocyte. Some Z1 zygotes from long protocol may originated from immature oocytes with poorer potential and therefore had poorer pregnancy outcomes than Z1 zygotes from GnRH-ant protocol. The findings may also explain why Z-score could not be consistently verified in previous studies which used long protocol. There has been debate regarding the use of the Z-score for predicting outcomes following assisted reproduction [9, 25, 41–49]. A recent systematic review [18] reported no conclusive data on the clinical efficacy of the Z-score in fresh cycles, even though biological results showed a good relationship with embryo viability and suggest a role in cycles with day-1 transfer/freezing. These inconsistent results may have occurred because almost all of these studies used the long protocol rather than GnRH-ant. With GnRH-ant, the number of Z1 in the transferred cohort of embryos was significantly correlated with the clinical pregnancy rate and live birth rate in our study (*r* = 0.971; *p* = 0.029). However, this correlation was not observed for the long protocol. A possible explanation for the Z-score having better predictive value in the GnRH-ant protocol is that zygotes from the GnRH-ant protocol were taken from a more natural pattern of follicle recruitment, while the long protocol only improved the zygotes’ morphology without changing the survival potential.

The present study has some limitations. First, this retrospective study has risk of selection bias. A matched control group might improve the quality of the findings. Second, the relationship between pregnancy outcomes and Z-score for single embryo transfer requires further investigation. A mean of approximately two embryos were transferred to most patients in this study, so single-embryo outcomes could not be compared with the Z-score. Third, the timing of assessment is critical, as pronuclear development is a dynamic process. Thus, determination of the Z-score from a single light microscopy observation should be used with caution and only in conjunction with other methods of evaluation [27].

In conclusion, the Z-score distribution was poorer for the GnRH-ant protocol than the long protocol. This may be because these protocols have different effects on the endocrine profile or GnRH receptors. Additionally, the Z-score is a more feasible parameter for predicting embryo viability in IVF/ICSI with the GnRH-ant protocol than the long protocol.

## Supporting information

S1 Fig Zygote scoring system of Scott et al.[21] Z1 zygotes have an equal number of nucleoli aligned at the PN junctions (A). Z2 zygotes have an equal number and size of nucleoli (3 to 7) that are equally scattered in the 2 PNs(B) Z3 zygotes are characterized by inequality of the nuclei (unequal size, unequal numbers, or unequal alignment at the PN junction)(C and D). Z4 zygotes have PN that are separated or different in size and small nucleoli that are partially aligned or scattered.(E and F)
(TIF)

## References

[1] European, Middle East Orgalutran Study G. Comparable clinical outcome using the GnRH antagonist ganirelix or a long protocol of the GnRH agonist triptorelin for the prevention of premature LH surges in women undergoing ovarian stimulation. Human reproduction. 2001;16(4):644–51. 11278211 10.1093/humrep/16.4.644

[2] Huirne JA, Lambalk CB. Gonadotropin-releasing-hormone-receptor antagonists. Lancet. 2001;358(9295):1793–803. 10.1016/S0140-6736(01)06797-6 11734258

[3] Olivennes F, Frydman R. Friendly IVF: the way of the future? Human reproduction. 1998;13(5):1121–4. 9647528 10.1093/humrep/13.5.1121

[4] Xiao JS, Su CM, Zeng XT. Comparisons of GnRH antagonist versus GnRH agonist protocol in supposed normal ovarian responders undergoing IVF: a systematic review and meta-analysis. PloS one. 2014;9(9):e106854 PubMed Central PMCID: PMC4162565. 10.1371/journal.pone.0106854 25216031 PMC4162565

[5] Al-Inany HG, Youssef MA, Ayeleke RO, Brown J, Lam WS, Broekmans FJ. Gonadotrophin-releasing hormone antagonists for assisted reproductive technology. The Cochrane database of systematic reviews. 2016;4:CD001750 10.1002/14651858.CD001750.pub4 27126581 PMC8626739

[6] Orvieto R, Patrizio P. GnRH agonist versus GnRH antagonist in ovarian stimulation: an ongoing debate. Reproductive biomedicine online. 2013;26(1):4–8. 10.1016/j.rbmo.2012.11.001 23186555

[7] Grow D, Kawwass JF, Kulkarni AD, Durant T, Jamieson DJ, Macaluso M. GnRH agonist and GnRH antagonist protocols: comparison of outcomes among good-prognosis patients using national surveillance data. Reproductive biomedicine online. 2014;29(3):299–304. 10.1016/j.rbmo.2014.05.007 25043892

[8] Bosch E, Labarta E, Kolibianakis E, Rosen M, Meldrum D. Regimen of ovarian stimulation affects oocyte and therefore embryo quality. Fertility and sterility. 2016;105(3):560–70. 10.1016/j.fertnstert.2016.01.022 26826273

[9] Scott L. The biological basis of non-invasive strategies for selection of human oocytes and embryos. Human reproduction update. 2003;9(3):237–49. 12859045 10.1093/humupd/dmg023

[10] Senn A, Urner F, Chanson A, Primi MP, Wirthner D, Germond M. Morphological scoring of human pronuclear zygotes for prediction of pregnancy outcome. Human reproduction. 2006;21(1):234–9. 10.1093/humrep/dei282 16126750

[11] Zollner U, Zollner KP, Steck T, Dietl J. Pronuclear scoring. Time for international standardization. The Journal of reproductive medicine. 2003;48(5):365–9. 12815911

[12] Lan KC. The predictive value of using a combined Z-score and day 3 embryo morphology score in the assessment of embryo survival on day 5. Human reproduction. 2003;18(6):1299–306. 12773463 10.1093/humrep/deg239

[13] Zamora RB, Sanchez RV, Perez JG, Diaz RR, Quintana DB, Bethencourt JC. Human zygote morphological indicators of higher rate of arrest at the first cleavage stage. Zygote. 2011;19(4):339–44. 10.1017/S0967199410000407 20663238

[14] Maille L, Bergere M, Lemoine E, Camier B, Prevost JF, Bourdrel JM, et al Pronuclear morphology differs between women more than 38 and women less than 30 years of age. Reproductive biomedicine online. 2009;18(3):367–73. 19298736 10.1016/s1472-6483(10)60095-8

[15] Nicoli A, Valli B, Di Girolamo R, Di Tommaso B, Gallinelli A, La Sala GB. Limited importance of pre-embryo pronuclear morphology (zygote score) in assisted reproduction outcome in the absence of embryo cryopreservation. Fertility and sterility. 2007;88(4 Suppl):1167–73. 10.1016/j.fertnstert.2007.01.066 17467704

[16] Aydin S. Is pronuclear scoring a really good predictor for ICSI cycles? Gynecological endocrinology: the official journal of the International Society of Gynecological Endocrinology. 2011;27(10):742–7. 20807168 10.3109/09513590.2010.509829

[17] Weitzman VN, Schnee-Riesz J, Benadiva C, Nulsen J, Siano L, Maier D. Predictive value of embryo grading for embryos with known outcomes. Fertility and sterility. 2010;93(2):658–62. 10.1016/j.fertnstert.2009.02.032 19410247

[18] Nicoli AP, Capodanno S., Fini F., Falbo M., La Sala A., G. B. Pronuclear morphology evaluation for fresh in vitro fertilization (IVF) and intracytoplasmic sperm injection (ICSI) cycles: a systematic review. Journal of ovarian research. 2013;6(1):64 PubMed Central PMCID: PMC3847610. 10.1186/1757-2215-6-64 24028277 PMC3847610

[19] Tsai YR, Huang FJ, Lin PY, Kung FT, Lin YJ, Lin YC, et al Progesterone elevation on the day of human chorionic gonadotropin administration is not the only factor determining outcomes of in vitro fertilization. Fertility and sterility. 2015;103(1):106–11. 10.1016/j.fertnstert.2014.10.019 25455869

[20] Lin YC, Chang SY, Lan KC, Huang HW, Chang CY, Tsai MY, et al Human oocyte maturity in vivo determines the outcome of blastocyst development in vitro. Journal of assisted reproduction and genetics. 2003;20(12):506–12. PubMed Central PMCID: PMC3455306. 10.1023/B:JARG.0000013651.37866.0c 15035550 PMC3455306

[21] Scott L, Alvero R, Leondires M, Miller B. The morphology of human pronuclear embryos is positively related to blastocyst development and implantation. Human reproduction. 2000;15(11):2394–403. 11056141 10.1093/humrep/15.11.2394

[22] Veeck LL. Atlas of the human oocyte and early conceptus 1st ed Baltimore:Williams & Wilkins 1986.

[23] Zegers-Hochschild F, Adamson GD, de Mouzon J, Ishihara O, Mansour R, Nygren K, et al The International Committee for Monitoring Assisted Reproductive Technology (ICMART) and the World Health Organization (WHO) Revised Glossary on ART Terminology, 2009. Human reproduction. 2009;24(11):2683–7. 10.1093/humrep/dep343 19801627

[24] Rosner. Estimation of Survival Curves:The Kaplan-Meier Estimator 2010 761–73. p.

[25] Papale LF, A.;Montag M.;Tomasi G.;. The zygote. Human reproduction. 2012;27 Suppl 1:i22–49. 22811310 10.1093/humrep/des205

[26] Ajduk A, Ilozue T, Windsor S, Yu Y, Seres KB, Bomphrey RJ, et al Rhythmic actomyosin-driven contractions induced by sperm entry predict mammalian embryo viability. Nature communications. 2011;2:417 PubMed Central PMCID: PMC3265380. 10.1038/ncomms1424 21829179 PMC3265380

[27] Alpha Scientists in Reproductive M, Embryology ESIGo. The Istanbul consensus workshop on embryo assessment: proceedings of an expert meeting. Human reproduction. 2011;26(6):1270–83. 10.1093/humrep/der037 21502182

[28] Yanez LZ, Han J, Behr BB, Reijo Pera RA, Camarillo DB. Human oocyte developmental potential is predicted by mechanical properties within hours after fertilization. Nature communications. 2016;7:10809 PubMed Central PMCID: PMC4770082. 10.1038/ncomms10809 26904963 PMC4770082

[29] Gianaroli L, Magli MC, Ferraretti AP, Lappi M, Borghi E, Ermini B. Oocyte euploidy, pronuclear zygote morphology and embryo chromosomal complement. Human reproduction. 2007;22(1):241–9. 10.1093/humrep/del334 16936301

[30] Gianaroli L, Magli MC, Ferraretti AP, Fortini D, Grieco N. Pronuclear morphology and chromosomal abnormalities as scoring criteria for embryo selection. Fertility and sterility. 2003;80(2):341–9. 12909497 10.1016/s0015-0282(03)00596-x

[31] Depalo R, Lorusso F, Palmisano M, Bassi E, Totaro I, Vacca M, et al Follicular growth and oocyte maturation in GnRH agonist and antagonist protocols for in vitro fertilisation and embryo transfer. Gynecological endocrinology: the official journal of the International Society of Gynecological Endocrinology. 2009;25(5):328–34. 19340626 10.1080/09513590802617762

[32] Depalo R, Jayakrishan K, Garruti G, Totaro I, Panzarino M, Giorgino F, et al GnRH agonist versus GnRH antagonist in in vitro fertilization and embryo transfer (IVF/ET). Reproductive biology and endocrinology: RB&E. 2012;10:26. PubMed Central PMCID: PMC3442989. 22500852 10.1186/1477-7827-10-26 PMC3442989

[33] Khalaf M, Mittre H, Levallet J, Hanoux V, Denoual C, Herlicoviez M, et al GnRH agonist and GnRH antagonist protocols in ovarian stimulation: differential regulation pathway of aromatase expression in human granulosa cells. Reproductive biomedicine online. 2010;21(1):56–65. 10.1016/j.rbmo.2010.03.017 20457540

[34] Lin Y, Kahn JA, Hillensjo T. Is there a difference in the function of granulosa-luteal cells in patients undergoing in-vitro fertilization either with gonadotrophin-releasing hormone agonist or gonadotrophin-releasing hormone antagonist? Human reproduction. 1999;14(4):885–8. 10221213 10.1093/humrep/14.4.885

[35] Brus L, Lambalk CB, de Koning J, Helder MN, Janssens RM, Schoemaker J. Specific gonadotrophin-releasing hormone analogue binding predominantly in human luteinized follicular aspirates and not in human pre-ovulatory follicles. Human reproduction. 1997;12(4):769–73. 9159440 10.1093/humrep/12.4.769

[36] Cheung LW, Wong AS. Gonadotropin-releasing hormone: GnRH receptor signaling in extrapituitary tissues. The FEBS journal. 2008;275(22):5479–95. 10.1111/j.1742-4658.2008.06677.x 18959738

[37] Attaman J, Arbogast LK, Friedman CI, Danforth DR. Effect of gonadotropin-releasing hormone antagonist on primordial follicle survival in the primate ovary. The Journal of reproductive medicine. 2014;59(3–4):103–9. 24724216

[38] Rienzi L, Ubaldi FM, Iacobelli M, Minasi MG, Romano S, Ferrero S, et al Significance of metaphase II human oocyte morphology on ICSI outcome. Fertility and sterility. 2008;90(5):1692–700. 10.1016/j.fertnstert.2007.09.024 18249393

[39] Murber A, Fancsovits P, Ledo N, Gilan ZT, Rigo J Jr., Urbancsek J. Impact of GnRH analogues on oocyte/embryo quality and embryo development in in vitro fertilization/intracytoplasmic sperm injection cycles: a case control study. Reproductive biology and endocrinology: RB&E. 2009;7:103. PubMed Central PMCID: PMC2762973. 19781070 10.1186/1477-7827-7-103 PMC2762973

[40] Cota AM, Oliveira JB, Petersen CG, Mauri AL, Massaro FC, Silva LF, et al GnRH agonist versus GnRH antagonist in assisted reproduction cycles: oocyte morphology. Reproductive biology and endocrinology: RB&E. 2012;10:33. PubMed Central PMCID: PMC3464873. 22540993 10.1186/1477-7827-10-33 PMC3464873

[41] Senn AU, Chanson F., Primi A., Wirthner M. P., Germond D., M. Morphological scoring of human pronuclear zygotes for prediction of pregnancy outcome. Human reproduction. 2006;21(1):234–9. 10.1093/humrep/dei282 16126750

[42] Arroyo GV, Santalo A., Barri J., P. N. Developmental prognosis for zygotes based on pronuclear pattern: usefulness of pronuclear scoring. Journal of assisted reproduction and genetics. 2007;24(5):173–81. PubMed Central PMCID: PMC3455056. 10.1007/s10815-006-9099-0 17318392 PMC3455056

[43] Payne JF, Raburn DJ, Couchman GM, Price TM, Jamison MG, Walmer DK. Relationship between pre-embryo pronuclear morphology (zygote score) and standard day 2 or 3 embryo morphology with regard to assisted reproductive technique outcomes. Fertility and sterility. 2005;84(4):900–9. 10.1016/j.fertnstert.2005.04.047 16213842

[44] Ludwig AK, Werner S, Diedrich K, Nitz B, Ludwig M. The value of pronuclear scoring for the success of IVF and ICSI-cycles. Archives of gynecology and obstetrics. 2006;273(6):346–54. 10.1007/s00404-005-0102-2 16333679

[45] Weitzman VN. Predictive value of embryo grading for embryos with known outcomes. Fertility and sterility. 2010;93(2):658–62. 10.1016/j.fertnstert.2009.02.032 19410247

[46] Aydin S, Cinar O, Demir B, Korkmaz C, Ozdegirmenci O, Dilbaz S, et al Is pronuclear scoring a really good predictor for ICSI cycles? Gynecological endocrinology: the official journal of the International Society of Gynecological Endocrinology. 2011;27(10):742–7. 20807168 10.3109/09513590.2010.509829

[47] Azzarello A, Hoest T, Mikkelsen AL. The impact of pronuclei morphology and dynamicity on live birth outcome after time-lapse culture. Human reproduction. 2012;27(9):2649–57. 10.1093/humrep/des210 22740496

[48] Braga DP, Setti AS, Figueira Rde C, Iaconelli A Jr., Borges E Jr. The combination of pronuclear and blastocyst morphology: a strong prognostic tool for implantation potential. Journal of assisted reproduction and genetics. 2013;30(10):1327–32. PubMed Central PMCID: PMC3824860. 10.1007/s10815-013-0073-3 23934020 PMC3824860

[49] Berger DS, Zapantis A, Merhi Z, Younger J, Polotsky AJ, Jindal SK. Embryo quality but not pronuclear score is associated with clinical pregnancy following IVF. Journal of assisted reproduction and genetics. 2014;31(3):279–83. PubMed Central PMCID: PMC3947071. 10.1007/s10815-013-0162-3 24390678 PMC3947071

