# Supplementary figures and images for: Reassessing the feasibility of the zygote score for predicting embryo viability in IVF/ICSI using the GnRH antagonist protocol compared to the long protocol
Source: PLoS One. 2017 Feb 2;12(2):e0171465. doi: 10.1371/journal.pone.0171465 (PMC5289632; doi:10.1371/journal.pone.0171465)

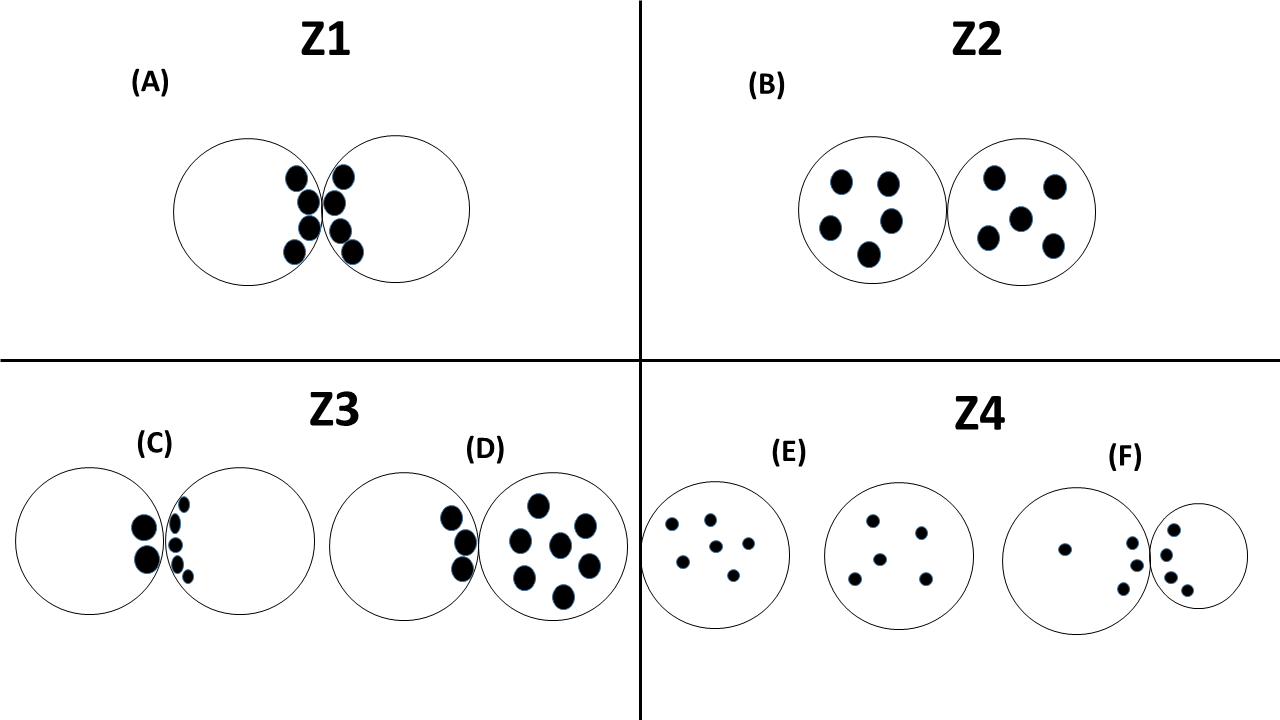

Supplement: S1 Fig — Zygote scoring system of Scott et al.[21] Z1 zygotes have an equal number of nucleoli aligned at the PN junctions (A). Z2 zygotes have an equal number and size of nucleoli (3 to 7) that are equally scattered in the 2 PNs(B) Z3 zygotes are characterized by inequality of the nuclei (unequal size, unequal numbers, or unequal alignment at the PN junction)(C and D). Z4 zygotes have PN that are separated or different in size and small nucleoli that are partially aligned or scattered.(E and F) (TIF) [file pone.0171465.s001.tif]
